# Case Report: Real-world experience with pacritinib after prior ruxolitinib exposure in myelofibrosis in Saudi Arabia: a report of five cases

**DOI:** 10.3389/fmed.2026.1950128

**Published:** 2026-10-26

**Authors:** Ayel Yahya, Ibraheem Motabi, Manal Aljohani, Khalid Abdullah Alghamdi, Walaa A. Rajkhan, Ahmad S. Alsaeed, Ashraf Warsi

**Affiliations:** 1 Department of Oncology, Division of Adult Haematology, King Abdulaziz Medical City, Riyadh, Saudi Arabia; 2 Hematology Department, King Fahad Medical City, Riyadh, Ministry of National Guard Health Affairs, Riyadh, Saudi Arabia; 3 Clinical Pharmacist, King Abdulaziz Medical City, Riyadh, Saudi Arabia; 4 Haematology Fellow, King Abdulaziz Medical City, Riyadh, Saudi Arabia; 5 Department of Adult Haematology/Bone Marrow Transplant Section, King Abdulaziz Medical City, Ministry of National Guard-Health Affairs, Jeddah, Saudi Arabia; 6 College of Medicine, King Saud Bin Abdulaziz University for Health Sciences, Jeddah, Saudi Arabia; 7 Department of Adult Haematology, King Abdulaziz Medical City (National Guard), Jeddah, Saudi Arabia

**Keywords:** JAK inhibitors, myelofibrosis, pacritinib, Saudi Arabia, thrombocytopenia

## Abstract

**Background:**

Treatment selection in myelofibrosis with cytopenic features remains challenging, particularly after prior ruxolitinib exposure. Pacritinib has been developed for patients with myelofibrosis and thrombocytopenia; however, real-world regional data remain limited. This case series describes pacritinib use in five patients with previously treated myelofibrosis in Saudi Arabia.

**Case presentation:**

Four patients had primary myelofibrosis, and one had post-polycythaemia vera myelofibrosis. All patients had prior ruxolitinib exposure, which had been discontinued because of disease progression or adverse events. At pacritinib initiation, the cohort showed clinically advanced disease features, including severe thrombocytopenia in three patients, anaemia in three patients, clinically significant splenomegaly in all patients, and marked leukocytosis in two patients. Pacritinib was initiated at 200 mg orally twice daily in all cases; one patient required dose reduction to 100 mg twice daily after a marked decline in white blood cell count, with an absolute neutrophil count of 1.2 × 10⁹/L. At the last available assessment, platelet counts and haemoglobin levels were numerically higher than baseline in all five patients, and palpable spleen length decreased from baseline in all patients with available follow-up spleen assessment. In one transfusion-dependent patient, red blood cell transfusion requirement decreased from approximately twice weekly before pacritinib initiation to once every 2 weeks during follow-up. One patient had no documented adverse events. Among the remaining four patients, documented non-haematologic adverse events were grade 1–2 and included fatigue, nausea with abdominal pain, and mild diarrhoea; one patient also had a marked leukocyte decline that prompted dose reduction.

**Conclusion:**

This case series describes pacritinib use after prior ruxolitinib exposure in selected patients with myelofibrosis, including patients with severe thrombocytopenia and concomitant anaemia. The observed changes in haematologic parameters and palpable spleen size are descriptive and do not establish treatment efficacy. Prospective regional studies with standardised response assessment, systematic safety collection, and longer follow-up are needed.

## Introduction

Myelofibrosis is a rare disorder affecting approximately 1 in 500,000 people worldwide (1). In the Middle East, the prevalence and incidence of myeloproliferative neoplasms (MPNs) are estimated to be 57–81 and 12–15 per 100,000 hospital patients per year, respectively (2). Myelofibrosis is classified as an MPN and is characterised by dysregulation in Janus kinase (JAK)/signal transducers and activators of transcription (STAT) signalling and excessive production of inflammatory cytokines (3). In advanced stages, the disease presents with progressive bone marrow fibrosis, cytopenias often necessitating red blood cell (RBC) and/or platelet transfusions, splenomegaly, and severe systemic symptoms (4). The median survival for affected patients is approximately 6–7 years (5). Thrombocytopenia is a negative prognostic factor that becomes more prevalent as the disease progresses, correlating with a higher symptom burden and reduced survival (6). Myelofibrosis management typically focuses on alleviating symptoms, reducing splenomegaly, and addressing cytopenias, with treatment strategies often including supportive care, cytoreductive therapies, and bone marrow transplantation in eligible patients (7).

The introduction of JAK inhibitors has marked a significant advancement, providing symptom control and spleen volume reduction (SVR); however, their use is often limited by adverse effects, including treatment-emergent or worsening cytopenias, and resistance or intolerance among certain patient groups (8). Pacritinib, a JAK2/FMS-like tyrosine kinase 3 (FLT3) inhibitor with minimal JAK1 activity, targets the interleukin-1 inflammatory pathway through inhibition of interleukin-1 receptor-associated kinase 1 (IRAK1) (9). This therapeutic agent has demonstrated clinical efficacy in myelofibrosis patients with limited myelosuppression. In the phase 3 PERSIST-1 trial, pacritinib, administered once daily, was compared with best available therapy (BAT), excluding ruxolitinib, and showed substantial and lasting reductions in spleen volume and symptom control, irrespective of baseline platelet levels (10). In the phase 3 PERSIST-2 trial, pacritinib was compared to BAT, including ruxolitinib, in patients with myelofibrosis and platelet counts of 100 × 10⁹/L or less. In the overall pacritinib-treated population, a ≥ 35% SVR was achieved in 18% of patients compared with 3% in the BAT group (*P* = 0.001). A clinically meaningful reduction (≥50%) in total symptom score was achieved in 25% of patients receiving pacritinib and 14% receiving BAT; however, this between-group difference did not reach statistical significance (*P* = 0.08) (11).

Based on these findings, the U.S. Food and Drug Administration (FDA) granted accelerated approval to pacritinib for the treatment of adults with intermediate or high-risk primary or secondary myelofibrosis who have a platelet count below 50 × 10⁹/L. The recommended dose is 200 mg orally twice daily. Nonetheless, limited real-world data exist regarding the clinical course and safety of pacritinib after prior JAK-inhibitor exposure. This report describes a multicentre, real-world experience with pacritinib in five patients with previously treated myelofibrosis in Saudi Arabia.

## Methods

This multicentre retrospective case series included adult patients with primary or secondary myelofibrosis who had prior ruxolitinib exposure and subsequently received pacritinib as part of routine clinical practice at participating centres in Saudi Arabia. Eligible patients were identified through haematology outpatient clinics and collaborating centres using available clinical records and treating-physician referral. Clinical data were abstracted at pacritinib initiation (baseline) and at approximately 3 and 6 months, corresponding to assessment windows of 3 months (±2 weeks) and 6 months (±2 weeks). Extracted variables included age and sex, myelofibrosis subtype and risk status, JAK2 V617F status, bone marrow fibrosis grade, prior myelofibrosis-directed therapy, pacritinib dose and dose modifications, haemoglobin, white blood cell and platelet counts, and palpable spleen length in centimetres. Where available, spleen volume, MPN-SAF TSS 2.0 scores, peripheral blood blasts, red blood cell transfusion requirements, concomitant medications, treatment status, and adverse events were also recorded. Findings were summarized descriptively because the available data did not permit systematic application of standardized myelofibrosis response criteria. Safety information was derived from adverse events and treatment modifications documented in the available clinical records.

## Case presentation

Five patient cases are presented. All patients had prior JAK-inhibitor exposure and were initiated on pacritinib at 200 mg orally twice daily. Baseline characteristics and treatment exposure are summarized in Table 1.

**Table 1:** Clinical characteristics and pacritinib exposure across five cases.

Case	Age/sex	Myelofibrosis subtype	Risk status/ECOG	JAK2 V617F status	Baseline fibrosis grade	Prior myelofibrosis-directed therapy	Pacritinib exposure
Case 1	54/M	Primary myelofibrosis	DIPSS intermediate-2	Negative	MF-2	Two prior lines: ruxolitinib 10 mg once daily, discontinued because of intolerance (fatigue, dizziness, and headache); lenalidomide 10 mg once daily plus steroids, discontinued because of disease progression	200 mg orally twice daily; no dose reduction reported
Case 2	74/F	Primary myelofibrosis	DIPSS intermediate-1; ECOG 1	Negative	MF-2	Three prior lines: hydroxycarbamide, discontinued because of disease progression; ruxolitinib 5 mg daily for 1 week, discontinued because of adverse event; pegylated interferon, discontinued because of disease progression with no clinical improvement	Initiated on 24 March 2024 at 200 mg orally twice daily; reduced to 100 mg twice daily on 16 October 2024 following marked leukocyte decline; ANC 1.2 × 10⁹/L
Case 3	69/F	Post-polycythaemia vera myelofibrosis	NR	Positive	MF-3	One prior line: ruxolitinib 10 mg daily for 2 years, discontinued because of an adverse event	Initiated on 1 July 2025 at 200 mg orally twice daily; no dose reduction reported; patient remained on pacritinib at last follow-up
Case 4	66/M	Primary myelofibrosis	DIPSS intermediate-1	Negative	MF-1	One prior line: ruxolitinib 5 mg daily, discontinued because of disease progression	Initiated in August 2024 at 200 mg orally twice daily; no dose reduction reported; patient remained on pacritinib at last follow-up
Case 5	70/M	Primary myelofibrosis	NR	Positive	NR	One prior line: ruxolitinib 10 mg daily, discontinued because of disease progression	Initiated in May 2025 at 200 mg orally twice daily; no dose reduction reported

ANC, Absolute Neutrophil Count; DIPSS, Dynamic International Prognostic Scoring System; ECOG, Eastern Cooperative Oncology Group; MPN-SAF TSS, Myeloproliferative Neoplasm Symptom Assessment Form Total Symptom Score; NR, not reported.

### Case 1

A 54-year-old Saudi man with primary myelofibrosis had intermediate-2 risk disease by the Dynamic International Prognostic Scoring System (DIPSS) at the time of pacritinib initiation. Molecular testing was negative for JAK2 V617F. Baseline assessment showed marked anaemia, with a haemoglobin level of 67 g/L, a white blood cell count of 2.83 × 10⁹/L, and a platelet count of 175 × 10⁹/L (Tables 1, 2). Palpable splenomegaly was documented at 28 cm, and bone marrow fibrosis was reported as MF-2. The patient was transfusion-dependent before pacritinib initiation, with a red blood cell transfusion requirement of approximately twice weekly.

**Table 2:** Longitudinal laboratory and spleen measurements during pacritinib treatment.

Case	Baseline	Month 3	Month 6/last recorded follow-up
Case 1	Hb 67 g/L; WBC 2.83 × 10⁹/L; platelet count 175 × 10⁹/L; spleen length 28 cm; transfusion requirement approximately twice weekly	Hb 71 g/L; WBC 6.7 × 10⁹/L; platelet count 115 × 10⁹/L; spleen length 19.4 cm; patient remained transfusion-dependent and alive	Hb 87 g/L; WBC 5.83 × 10⁹/L; platelet count 176 × 10⁹/L; transfusion requirement decreased to once every 2 weeks; patient alive at last follow-up
Case 2	MPN-SAF TSS 18; Hb 117 g/L; WBC 30.1 × 10⁹/L; platelet count 39 × 10⁹/L; spleen length 18.3 cm; spleen volume 460 cm^3^	MPN-SAF TSS 10; Hb 117 g/L; WBC 25.76 × 10⁹/L; platelet count 41 × 10⁹/L; spleen length 19 cm; peripheral blasts remained 0 to <5%	Hb 130 g/L; WBC 3.87× 10⁹/L; platelet count 47 × 10⁹/L; spleen length 15 cm; fatigue, fever, night sweats, and abdominal pain improved; patient alive at last follow-up
Case 3	Hb 119 g/L; WBC 4.84 × 10⁹/L; platelet count 46 × 10⁹/L; spleen length 24 cm	Hb 103 g/L; WBC 4.49 × 10⁹/L; platelet count 49 × 10⁹/L; spleen length 22 cm	Hb 120 g/L; WBC 3.87× 10⁹/L; platelet count 56 × 10⁹/L; spleen length 14 cm; patient alive and still receiving pacritinib at last follow-up
Case 4	Hb 83 g/L; WBC 6.59 × 10⁹/L; platelet count 108 × 10⁹/L; spleen length 21 cm	Hb 90 g/L; WBC 4.89 × 10⁹/L; platelet count 121 × 10⁹/L	Hb 102 g/L; WBC 5.75 × 10⁹/L; platelet count 124 × 10⁹/L; spleen length 19 cm; patient alive and still receiving pacritinib at last follow-up
Case 5	Hb 86 g/L; WBC 96.93 × 10⁹/L; platelet count 30 × 10⁹/L; spleen length 24.5 cm	Hb 97 g/L; WBC 106 × 10⁹/L; platelet count 55 × 10⁹/L; spleen length 28 cm	Hb 110 g/L; WBC 91.3 × 10⁹/L; platelet count 54 × 10⁹/L; spleen length 18 cm

Hb, haemoglobin; NR, not reported; WBC, white blood cell count.

Before pacritinib, the patient had received two prior lines of therapy. Ruxolitinib 10 mg once daily was used as first-line treatment and was discontinued because of intolerance characterised by fatigue, dizziness, and headache. Second-line treatment consisted of lenalidomide 10 mg once daily plus steroids, which was also discontinued because of disease progression. Although the baseline platelet count of 175 × 10⁹/L was above the platelet threshold in the FDA-approved indication described above, pacritinib was selected because of ruxolitinib intolerance and substantial symptomatic splenomegaly that was affecting daily activities. Pacritinib was initiated at a dose of 200 mg orally twice daily, and no dose reduction was reported during follow-up.

At the 3-month assessment, palpable spleen length had decreased from 28 cm at baseline to 19.4 cm. The platelet count decreased from 175 × 10⁹/L to 115 × 10⁹/L, whereas the white blood cell count increased from 2.83 × 10⁹/L to 6.7 × 10⁹/L and haemoglobin increased from 67 g/L to 71 g/L. The patient remained transfusion-dependent and was alive at this time point. At the 6-month assessment, the platelet count had recovered to 176 × 10⁹/L, the white blood cell count was 5.83 × 10⁹/L, and haemoglobin had increased to 87 g/L. The patient remained transfusion-dependent; however, the transfusion requirement decreased from approximately twice weekly before pacritinib initiation to once every 2 weeks during follow-up. The patient was alive at the last recorded follow-up.

### Case 2

A 74-year-old Saudi woman with primary myelofibrosis had intermediate-1 risk disease by the DIPSS and an Eastern Cooperative Oncology Group (ECOG) performance status of 1 at pacritinib initiation. Molecular testing was negative for JAK2 V617F. Baseline disease assessment showed Myeloproliferative Neoplasm Symptom Assessment Total Symptom Score (MPN-SAF TSS) 2.0 score of 18, palpable splenomegaly of 18.3 cm, spleen volume of 460 cm^3^, and bone marrow fibrosis grade MF-2. Laboratory evaluation showed thrombocytopenia, with a platelet count of 39 × 10⁹/L, leukocytosis, with a white blood cell count of 30.1 × 10⁹/L, and haemoglobin of 117 g/L (Tables 1, 2). Peripheral blasts were in the 0 to <5% category. Relevant comorbidities included type 2 diabetes mellitus and dyslipidaemia, and concomitant medications included antidiabetic treatment and lipid-lowering therapy.

Before pacritinib, she had received three prior lines of therapy. Hydroxycarbamide was used as first-line treatment and was discontinued because of disease progression. She subsequently received ruxolitinib 5 mg daily for 1 week, which was discontinued because of an adverse event. Pegylated interferon was administered as third-line therapy and was discontinued because of disease progression, with no clinical improvement. Pacritinib was initiated on 24 March 2024 at 200 mg orally twice daily. On 16 October 2024, the dose was reduced to 100 mg twice daily following a marked reduction in white blood cell count during follow-up; the absolute neutrophil count was 1.2 × 10⁹/L. No clinically significant infection was reported, and the dose adjustment was made as a precautionary measure to mitigate the risk of treatment-related cytopenia.

At the 3-month assessment, the MPN-SAF TSS 2.0 score had decreased from 18 at baseline to 10. Palpable spleen length increased slightly from 18.3 cm to 19 cm. The platelet count remained stable, increasing from 39 × 10⁹/L to 41 × 10⁹/L, whereas the white blood cell count decreased from 30.1 × 10⁹/L to 25.76 × 10⁹/L. Haemoglobin remained unchanged at 117 g/L. Peripheral blasts remained in the 0 to <5% category.

At the 6-month assessment, palpable spleen length had decreased to 15 cm, indicating a reduction from both baseline and the 3-month assessment. The platelet count increased to 47 × 10⁹/L, the white blood cell count was 3.87 × 10⁹/L, and haemoglobin increased to 130 g/L. Clinical symptoms, including fatigue, fever, night sweats, and abdominal pain, were reported to have improved during follow-up. The patient was alive at the last recorded follow-up.

### Case 3

A 69-year-old Saudi woman was diagnosed with JAK2 V617F-positive post-polycythaemia vera myelofibrosis at the age of 56 years. Before pacritinib, the patient had received ruxolitinib 10 mg daily as first-line treatment for 2 years, which was discontinued because of an adverse event. Pacritinib was initiated on 1 July 2025 at a dose of 200 mg orally twice daily. No dose reduction was reported during follow-up, and the patient remained on pacritinib at the last recorded follow-up. At pacritinib initiation, she had palpable splenomegaly of 24 cm and bone marrow fibrosis grade MF-3. Laboratory evaluation showed thrombocytopenia, with a platelet count of 46 × 10⁹/L, a white blood cell count of 4.84 × 10⁹/L, and a haemoglobin level of 119 g/L (Tables 1, 2).

At the 3-month assessment, palpable spleen length had decreased modestly from 24 cm at baseline to 22 cm. The platelet count increased from 46 × 10⁹/L to 49 × 10⁹/L, whereas the white blood cell count remained stable at 4.49 × 10⁹/L. Haemoglobin decreased from 119 g/L to 103 g/L. At the 6-month assessment, palpable spleen length had decreased further to 14 cm. The platelet count increased to 56 × 10⁹/L, the white blood cell count was 3.87 × 10⁹/L, and haemoglobin increased to 120 g/L. The patient was alive and remained on pacritinib at the last recorded follow-up.

### Case 4

A 66-year-old Saudi man with primary myelofibrosis was diagnosed at the age of 54 years, on 1 April 2015, with a disease duration of approximately 131 months before data collection. At pacritinib initiation, the patient was 64 years old and had intermediate-1 risk disease according to the DIPSS. Molecular testing was negative for JAK2 V617F. Baseline assessment showed anaemia, with a haemoglobin level of 83 g/L, a white blood cell count of 6.59 × 10⁹/L, and a platelet count of 108 × 10⁹/L (Tables 1, 2). Palpable splenomegaly was documented at 21 cm, and reticulin and collagen fibrosis staging was reported as MF-1.

Before pacritinib initiation, the patient had received one prior line of myelofibrosis-directed therapy. Ruxolitinib was used as first-line treatment at a dose of 5 mg daily and was discontinued because of disease progression. Pacritinib was initiated in August 2024 at a dose of 200 mg orally twice daily. No dose reduction or reduced-dose schedule was reported during follow-up, and the patient remained on pacritinib at the last recorded follow-up.

At the 3-month assessment, the platelet count increased from 108 × 10⁹/L at baseline to 121 × 10⁹/L, whereas the white blood cell count decreased from 6.59 × 10⁹/L to 4.89 × 10⁹/L. Haemoglobin increased from 83 g/L to 90 g/L. At the 6-month assessment, palpable spleen length had decreased from 21 cm at baseline to 19 cm. The platelet count increased further to 124 × 10⁹/L, the white blood cell count was 5.75 × 10⁹/L, and haemoglobin increased to 102 g/L. The patient was alive and remained on pacritinib at the last recorded follow-up. Figure 1 shows the longitudinal haemoglobin and platelet count changes during pacritinib treatment across five cases.

**Figure 1:**
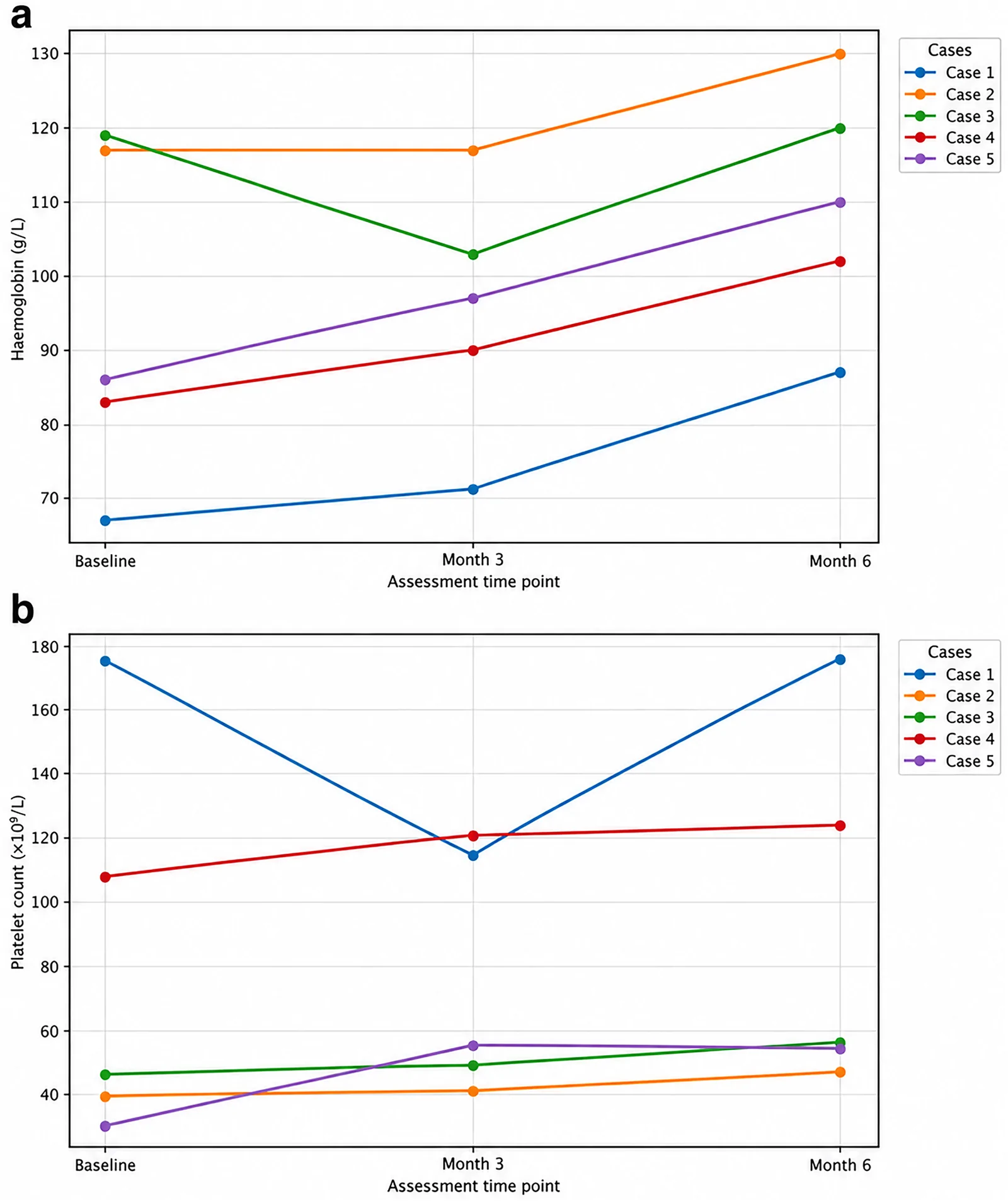
Longitudinal haemoglobin and platelet count changes during pacritinib treatment across five cases. **(a)** Haemoglobin values and **(b)** platelet counts are shown at baseline, month 3, and month 6 for each of the five cases treated with pacritinib.

### Case 5

A 70-year-old Saudi man with primary myelofibrosis was diagnosed in January 2024. Molecular testing was positive for JAK2 V617F. At baseline, the patient had substantial disease burden, characterised by marked leukocytosis, thrombocytopenia, anaemia, and clinically significant splenomegaly. Laboratory evaluation showed a platelet count of 30 × 10⁹/L, a white blood cell count of 96.93 × 10⁹/L, and a haemoglobin level of 86 g/L. Palpable spleen length was 24.5 cm (Tables 1, 2).

Before pacritinib, the patient had received one prior line of myelofibrosis-directed therapy. Ruxolitinib was used as first-line treatment at a dose of 10 mg daily and was discontinued because of disease progression. Pacritinib was initiated in May 2025 at a dose of 200 mg orally twice daily. No dose reduction was recorded during follow-up.

At the 3-month assessment, palpable spleen length had increased from 24.5 cm at baseline to 28 cm. The platelet count increased from 30 × 10⁹/L to 55 × 10⁹/L, and haemoglobin increased from 86 g/L to 97 g/L. The white blood cell count remained markedly elevated at 106 × 10⁹/L. At the 6-month assessment, palpable spleen length had decreased to 18 cm, indicating a reduction from both baseline and the 3-month assessment. The platelet count was 54 × 10⁹/L, the white blood cell count was 91.3 × 10⁹/L, and haemoglobin increased to 110 g/L. Compared with baseline, the platelet count and haemoglobin level were numerically higher, whereas leukocytosis persisted. Figure 2 shows the percent change from baseline at the last available follow-up across five cases.

**Figure 2:**
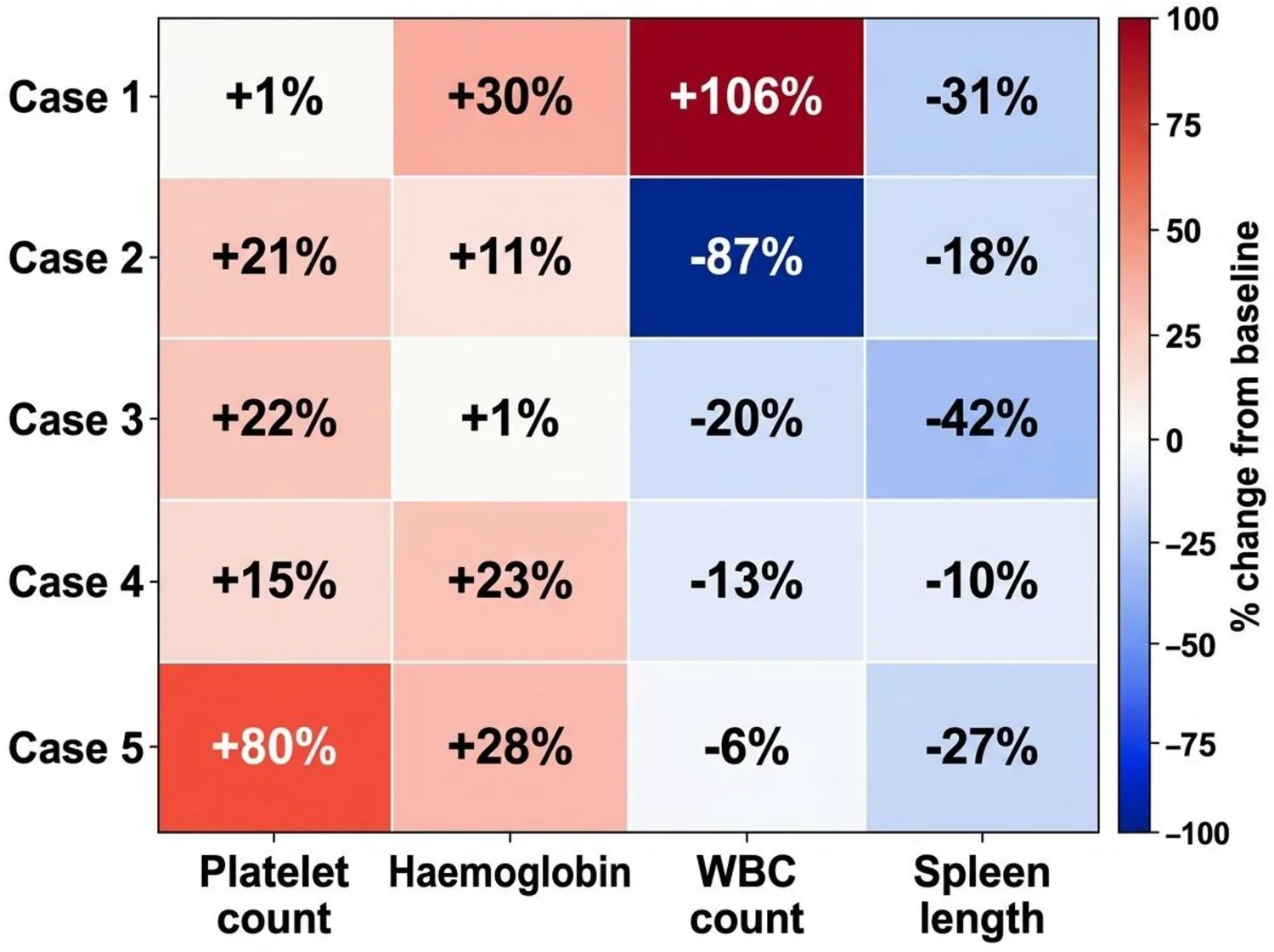
Percent change from baseline at last available follow-up across five cases**.** Heatmap showing the percent change from baseline to the last available follow-up in platelet count, haemoglobin, white blood cell (WBC) count, and palpable spleen length for each of the five cases treated with pacritinib.

### Safety and tolerability

Documented treatment-emergent adverse events were predominantly grade 1–2. Case 1 had grade 2 fatigue; Case 2 had grade 2 nausea and abdominal pain and also experienced a marked leukocyte decline that prompted pacritinib dose reduction, with an absolute neutrophil count of 1.2 × 10⁹/L; Cases 3 and 5 had grade 1 mild diarrhoea; and no adverse event was reported for Case 4 (Table 3). No clinically significant infection was reported in Case 2. Because safety ascertainment was retrospective and not systematic, these findings represent documented events and should not be interpreted as estimates of adverse-event incidence or as evidence of the absence of toxicity.

**Table 3:** Documented treatment-emergent adverse events during pacritinib treatment.

Case	Documented adverse event	CTCAE grade	Management/relevant safety information
Case 1	Fatigue	Grade 2	No pacritinib dose reduction reported
Case 2	Nausea and abdominal pain; marked leukocyte decline	Grade 2 for nausea/abdominal pain; haematologic event not prospectively graded	Pacritinib reduced from 200 mg to 100 mg twice daily on 16 October 2024; ANC 1.2 × 10⁹/L; no clinically significant infection reported
Case 3	Mild diarrhoea	Grade 1	No pacritinib dose reduction reported
Case 4	No adverse event reported	—	No pacritinib dose reduction reported
Case 5	Mild diarrhoea	Grade 1	No pacritinib dose reduction reported

ANC, absolute neutrophil count; CTCAE, Common Terminology Criteria for Adverse Events.

## Discussion

This case series describes a multicentre real-world experience with pacritinib in five patients with primary or post-polycythaemia vera myelofibrosis in Saudi Arabia. The series represents a clinically heterogeneous and treatment-challenging population, with prior ruxolitinib exposure in all five cases, severe thrombocytopenia in three cases, anaemia in three cases, clinically significant splenomegaly in all patients, and marked leukocytosis in two cases. Pacritinib was initiated at 200 mg orally twice daily in all cases. Four patients had no documented dose reduction during follow-up, whereas one patient underwent dose reduction to 100 mg twice daily after a marked leukocyte decline. At the last available follow-up, platelet count and haemoglobin were numerically higher than baseline in all five cases, and palpable spleen length decreased from baseline in all cases with available follow-up assessment. These findings are descriptive and should not be interpreted as evidence of treatment efficacy.

The baseline characteristics of this series reflect a clinically challenging population in which treatment options may be limited by cytopenias. Three of five patients had baseline platelet counts below 50 × 10⁹/L, and three had baseline haemoglobin below 100 g/L. Thrombocytopenia in myelofibrosis is an adverse disease feature and is associated with advanced disease biology, greater symptom burden, increased bleeding risk, and poorer survival (12). This context is relevant because treatment selection in thrombocytopenic myelofibrosis is often constrained by treatment-emergent or worsening cytopenias. In the present series, platelet count was maintained or numerically higher than baseline at the last available follow-up in all five patients, including the three patients with baseline severe thrombocytopenia. These observations describe laboratory changes only; causal inference is limited by the retrospective design, absence of a comparator group, and small case-series sample size.

Pacritinib is particularly relevant in this setting because its pharmacologic profile differs from earlier JAK-inhibitor options. It inhibits JAK2 and interleukin-1 receptor-associated kinase 1 (IRAK1), with limited JAK1 inhibition (9, 13). Although its development program supports use in patients with baseline cytopenias, this pharmacologic profile does not preclude treatment-related cytopenias; in the present series, Case 2 had a marked leukocyte decline with an absolute neutrophil count of 1.2 × 10⁹/L that prompted dose reduction. Emerging mechanistic and clinical data also indicate inhibition of activin A receptor type 1 (ACVR1), a pathway implicated in hepcidin regulation and anaemia biology in myelofibrosis (14). In the analysis by Oh et al., pacritinib demonstrated potent ACVR1 inhibition and was associated with higher rates of transfusion independence and reduction in transfusion burden compared with best available therapy among patients in PERSIST-2 who were not transfusion independent at baseline (14). These data provide a biological context for pacritinib use in patients with coexisting anaemia and thrombocytopenia, although the present case series was not designed to evaluate mechanistic or transfusion endpoints.

Palpable spleen measurements decreased from baseline to the last available assessment in all five cases. Case 1 showed a decrease from 28 to 19.4 cm at month 3, with no later spleen measurement available. At month 6, spleen length decreased from 18.3 to 15 cm in Case 2, from 24 to 14 cm in Case 3, from 21 to 19 cm in Case 4, and from 24.5 to 18 cm in Case 5. In Cases 2 and 5, spleen length increased at month 3 before decreasing by month 6. These changes cannot be classified as formal IWG-MRT/ELN spleen responses because follow-up CT/MRI-based spleen volume measurements were not available; the response framework requires imaging confirmation of at least 35% spleen volume reduction (15). In clinical trials, spleen volume reduction has been a major pacritinib endpoint. PERSIST-2 reported a higher spleen volume response rate with pacritinib than with best available therapy in patients with myelofibrosis and thrombocytopenia (11), and a pooled analysis in severe thrombocytopenia similarly reported higher rates of at least 35% spleen volume reduction with pacritinib (16). A *post hoc* landmark analysis of PERSIST-2 further suggested an association between spleen volume reduction and overall survival, although this remains hypothesis-generating (17).

Platelet trajectories were heterogeneous. Case 1 showed a transient decrease from 175 × 10⁹/L at baseline to 115 × 10⁹/L at month 3, followed by recovery to 176 × 10⁹/L at month 6. In Cases 2, 3, and 5, which had severe thrombocytopenia at baseline, platelet count changed from 39 to 47 × 10⁹/L, from 46 to 56 × 10⁹/L, and from 30 to 54 × 10⁹/L, respectively, by the last available assessment. Case 4 changed from 108 to 124 × 10⁹/L. These values are reported descriptively and should not be interpreted as a standardised platelet-response or as evidence of efficacy.

Anaemia was a major clinical issue at baseline across the case series. Haemoglobin was below 100 g/L in Cases 1, 4, and 5, and Case 1 was transfusion-dependent before pacritinib initiation. Anaemia in myelofibrosis may reflect marrow failure, inflammatory cytokine signalling, splenic sequestration, treatment effects, and transfusion-dependent disease evolution (4, 7). At the last available follow-up, haemoglobin was numerically higher than baseline in all five patients. Case 1 increased from 67 to 87 g/L, Case 4 from 83 to 102 g/L, and Case 5 from 86 to 110 g/L. In Case 3, haemoglobin decreased at month 3 and recovered to approximately baseline by month 6. Case 1 remained transfusion-dependent, although the reported transfusion frequency decreased from approximately twice weekly before pacritinib initiation to once every 2 weeks during follow-up. During pacritinib treatment, no erythropoiesis-stimulating agent, luspatercept, iron supplementation, androgen, corticosteroid, or other anaemia- or myelofibrosis-directed concomitant therapy was reported; red blood cell transfusion support was the relevant co-intervention in Case 1. Formal IWG-MRT/ELN anaemia response could not be assigned because the required transfusion-unit documentation and response-duration confirmation were incomplete (15).

All five patients had prior ruxolitinib exposure before pacritinib initiation, although recorded dose and duration varied substantially. Case 1 received ruxolitinib 10 mg once daily; Case 2 received 5 mg daily for 1 week and discontinued because of an adverse event; Case 3 received 10 mg daily for 2 years; Case 4 received 5 mg daily; and Case 5 received 10 mg daily. Outcomes after ruxolitinib discontinuation are generally poor, particularly among patients with cytopenias, advanced disease features, or inadequate response to prior JAK inhibition (18, 19). The PAC203 dose-finding study enrolled patients with advanced myelofibrosis who were resistant or intolerant to ruxolitinib and supported pacritinib 200 mg twice daily as the recommended dose based on clinical activity and tolerability (18). However, in the present series, the low or short-duration ruxolitinib exposure in some patients and the absence of documented formal resistance/refractoriness criteria preclude characterisation of the entire cohort as uniformly ruxolitinib-refractory. Therefore, the cohort is described as previously treated and ruxolitinib-exposed.

Documented safety events were generally low grade: grade 2 fatigue in Case 1, grade 2 nausea and abdominal pain in Case 2, and grade 1 mild diarrhoea in Cases 3 and 5; no adverse event was reported for Case 4. Case 2 additionally experienced a marked leukocyte decline with an absolute neutrophil count of 1.2 × 10⁹/L, prompting reduction of pacritinib to 100 mg twice daily; no clinically significant infection was reported. Four patients had no documented dose reduction, and no treatment discontinuation was recorded in the available follow-up. These observations should not be interpreted as establishing the incidence or overall tolerability of pacritinib because adverse events were retrospectively ascertained and were not systematically collected.

This case series has several limitations. First, the sample size was small and did not permit statistical analysis or causal inference. Second, the retrospective case-series design depended on available clinical documentation and may be susceptible to selection bias and variability in assessment timing. Third, safety ascertainment was retrospective rather than systematic; therefore, unrecorded adverse events cannot be excluded, and the absence of a documented event cannot be interpreted as a confirmed absence. Fourth, spleen assessment was based mainly on palpable spleen length rather than serial CT/MRI spleen volume, and serial symptom scores were largely unavailable, preventing standardised IWG-MRT/ELN spleen and symptom response assignment. Detailed transfusion-unit data and response-duration confirmation were also incomplete, preventing formal anaemia-response classification. Fifth, molecular characterisation was limited, and non-JAK2 mutations or high-molecular-risk profiles could not be evaluated. Sixth, follow-up was short, preventing assessment of response durability, leukemic transformation, long-term transfusion outcomes, treatment discontinuation, or survival. Finally, prior ruxolitinib exposure was heterogeneous in dose and duration, and formal criteria for ruxolitinib resistance/refractoriness were not documented for all cases.

In conclusion, this five-case real-world series from Saudi Arabia describes the use of pacritinib after prior ruxolitinib exposure in patients with myelofibrosis, including patients with severe thrombocytopenia, anaemia, splenomegaly, and heterogeneous prior treatment exposure. Documented adverse events were predominantly grade 1–2, although one patient required dose reduction after a marked leukocyte decline. These findings are descriptive and hypothesis-generating and should not be interpreted as evidence of efficacy or as a systematic assessment of safety. Prospective regional studies with standardised imaging-based spleen assessment, serial symptom scoring, transfusion-burden measurement, systematic adverse-event capture, and longer follow-up are needed.

## Data Availability

The datasets analysed during the current study are not publicly available due to confidentiality and data-protection requirements but may be made available upon reasonable request to the corresponding author, subject to institutional data-sharing approvals.
